# Low-dose decitabine-intensified modified conditioning regimen alleviates aGVHD in AML/MDS patients treated with allogeneic hematopoietic stem cell transplantation

**DOI:** 10.3389/fimmu.2023.1274492

**Published:** 2023-10-20

**Authors:** Jinye Zhu, Qingya Wang, Hanyun Ren, Yujun Dong, Yue Yin, Qian Wang, Zeyin Liang, Wei Liu, Qingyun Wang, Bingjie Wang, Yuan Li

**Affiliations:** Department of Hematology, Peking University First Hospital, Beijing, China

**Keywords:** low-dose decitabine, conditioning regimen, AML/MDS, allogeneic hematopoietic stem cell transplantation, graft versus host disease

## Abstract

**Background:**

The widespread adoption of Allogeneic Hematopoietic Stem Cell Transplantation (Allo-HSCT) has significantly improved the survival rates of patients with hematological malignancies. However, Graft-Versus-Host Disease (GVHD) remains a formidable complication, threatening patient prognosis. Recent research has indicated that decitabine (DAC), known for its hypomethylating properties may also exhibit immune-regulatory capabilities and a potential for reducing GVHD incidence and enhancing survival.

**Methods:**

We retrospectively reviewed data from AML/MDS patients who underwent Allo-HSCT at our center from January 2010 to January 2023. From a total of 251 patients with complete data, we employed propensity score matching (PSM) to create 100 matched pairs (200 patients) for comprehensive trial analysis. Patients receiving low-dose DAC-containing regimen were matched with those who did not receive DAC.

**Results:**

Patients in the DAC group exhibited a significantly lower incidence of grade II-IV acute GVHD (aGVHD) compared to non-DAC group (21% vs. 38%, P=0.013). Univariable and multivariable logistic regression analysis demonstrated DAC intervention as a protective factor against grade II-IV aGVHD (P=0.017, OR=0.47, 95% CI 0.23-0.81; P=0.018, OR=0.46, 95% CI 0.24-0.87). Multivariate competing risk regression further supported administration of decitabine as a protective factor against grade II-IV aGVHD (P=0.038, SHR=0.53, 95%CI 0.29-0.97). There was no significant difference between both groups concerning chronic GVHD, infection, disease relapse, overall survival, disease-free survival and GVHD free, relapse free survival. In MRD negative or intermediate risk subgroup, the grade II-IV aGVHD ameliorating effect of DAC was confirmed as well.

**Conclusion:**

Low-dose DAC-intensified modified conditioning regimen could improve prognosis in AML/MDS Patients treated with allogeneic hematopoietic stem cell transplantation.

## 1 Introduction

AML and MDS represent two major myeloid clonal diseases characterized by uncontrolled hematopoiesis, aggressive disease progression, and dismal prognosis. The natural course of AML and high-risk MDS is remarkably short, often less than six months. Epidemiological studies have reported a sobering 5-year survival rate of only 24-28.3% for AML patients, with median survival for higher-risk MDS patients being less than 3 years (1–3). Allogeneic hematopoietic stem cell transplantation (allo-HSCT) offers a potential cure for AML and intermediate and high-risk MDS patients, raising the 3-year overall survival to approximately 80% (4). However, the positive impact of transplantation is offset by post-transplantation complications, including graft versus disease (GVHD), disease relapse and virus reactivation, leading to a decline in the 5-year overall survival (OS) rate to around 30%. Among these complications, GVHD stands out as the most common and threatening, prompting extensive efforts in prevention and treatment. Nevertheless, the current consensus on GVHD management need to be refined and enhanced.

Hypomethylating agents (HMAs) have found extensive use in consolidation and bridging chemotherapy owing to their impact on hematologic malignancies through methylation regulation. Apart from their relative low toxicity and considerable effectiveness as antitumor therapy (5, 6), researchers have discovered their potential in epigenetically modulating immune responses (7). Notably, hypomethylating agents have been integrated into hematopoietic stem cell transplant protocols for AML/MDS treatment, resulting in significant progress (8–10). Among them, decitabine (DAC), a pyrimidine analogue, has drawn popular attention. A multicenter randomized control study demonstrated that hypomethylating drugs such as DAC could augment the graft versus leukemia (GVL) effect, thereby reducing the risk of relapse through the activation of natural killer (NK) cells and CD8+T cells (8). Additionally, a retrospective cohort trial revealed that incorporating decitabine into the traditional conditioning regimen could alleviate grade II-IV acute GVHD (aGVHD) and improve survival (11). However, a study from University of Wisconsin highlighted a potential association between decitabine administration and infection, which may influence the positive effects of DAC. Despite advancements in recent years, due to the limited scale of current studies, a consensus has not yet to be reached regarding the benefits of decitabine-containing regimens for AML/MDS patients undergoing HSCT. Moreover, variations in DAC dosage among studies have contributed to discrepancies in observations, making it challenging to evaluate the efficacy of DAC accurately.

In this study, our aim was to investigate the potential impact of incorporating low-dose decitabine into the conditioning regimen on the prognosis of patients undergoing allo-HSCT. To achieve this, we conducted a comprehensive retrospective analysis of data spanning over ten years from our center. Our analysis included an examination of baseline characteristics, engraftment, HSCT-related complications, and survival outcomes. The conclusive findings from our investigation demonstrated that a low-dose decitabine-containing regimen had a positive effect, alleviating acute GVHD and ultimately leading to improved patient prognosis.

## 2 Methods

### 2.1 Patients

Between January 2010 and January 2023, we conducted a retrospective data collection of AML/MDS patients who underwent allo-HSCT at the Bone Marrow Transplant Ward of Peking University First Hospital. From this pool of patients, we selected 251 individuals with relatively complete data. Among them, 125 patients received a conditioning regimen containing decitabine, while 126 patients did not receive decitabine as part of their regimen. To ensure balanced comparison between the two groups, we employed propensity score matching (PSM) analysis. Gender, age, diagnosis, donor type, conditioning regimen, and bone marrow blast count before transplantation were included in the matching process as covariates. A 1:1 matching ratio was applied, and the caliper width was set at 0.1. As a result, a final cohort of 200 patients was enrolled for the study. Ethical considerations were diligently observed throughout the study, which was conducted in accordance with the principles outlined in the Declaration of Helsinki. The Review Board of Peking University First Hospital provided approval for the study (No. 2023-432).

### 2.2 Conditioning regimen

The conditioning regimen for our study was determined based on either Bu/Cy or Bu/Flu, in accordance with the consensus on allogeneic hematopoietic stem cell transplantation for hematological diseases, as recommended by the Chinese Society of Hematology. Additionally, we took into account our institution’s previous protocol while formulating the conditioning approach for this research (4, 12). The concrete procedure was as follows: patients received cytarabine at a dose of 2g/m^2^/d intravenously for 3 days, busulfan at a dose of 3.2mg/kg/d intravenously for 3 days, and cyclophosphamide at a dose of 1.8g/m^2^/d intravenously for 2 days. Other than regimen based on Bu/Cy above, patients could receive Bu/Flu protocol including intravenous fludarabine at a total dose of 200mg/m^2^, and equal dose of Bu and cytarabine as the Bu/Cy protocol. Rabbit anti-thymocyte globulin (ATG) was administered intravenously 3 days before stem cell infusion as previous reported (11). For haploidentical transplant or MUD transplant, ATG was administrated as a total dose of 7.5mg/kg. For MSD donors with recipients age over 40 years, a total dose of 5mg/kg was administrated. Decitabine group additionally received intravenous decitabine at a dose of 15mg/m^2^ daily for 5 days before cytarabine administration.

### 2.3 GVHD prevention and treatment

The prevention protocol comprised cyclosporin A (CsA), mycophenolate mofetil (MMF), and short course methotrexate (MTX). The specific details were as follows: CsA was administered at a daily dose of 5mg/kg iv., starting 6 days before transplantation, aiming for an optimal serum trough concentration range of 150-250ng/mL. When digestive symptoms alleviated, CsA was taken orally. According to EBMT and Chinse consensus of allo-HSCT, CsA was usually administered for 3 months after HSCT and then gradually reduced to discontinuation in 6-9 months. While the course and dose should be adjusted dynamically according to the GVHD and relapse risk (13, 14). Mycophenolate mofetil (MMF) was initiated from the conditioning phase, given twice daily at a dose of 50mg iv. until 1 month post-transplantation. Additionally, on days +1, +3, +5, and +11, MTX was administered at a dose of 10-15mg/m^2^. Methylprednisolone served as routine therapy for GVHD, with a daily dose of 1-2 mg/kg. In the event of second-line alterations, basiliximab and ruxolitinib were utilized. As for the patients with positive FLT3-ITD mutation, FLT3 inhibitor such as sorafenib and gilteritinib was administrated.

### 2.4 Definitions and outcome measures

Patients in the study were diagnosed and classified based on the WHO classification of hematolymphoid tumors. For MDS, risk stratification was determined using the revised international prognostic scoring system (IPSS-R), while for AML, risk stratification was conducted following the European Leukemia Net (ELN) recommendations from the 2022 edition (15). The study cohort was divided into two categories: the “relative-intermediate” group, which included patients with IPSS-R scores ranging from 3.5 to 6, and AML patients with intermediate cytogenetic risk; and the “relative-high” group, consisting of patients with IPSS-R scores over 6 and AML patients with adverse cytogenetic risk. Monitoring of measurable residual disease (MRD) includes techniques such as multiparameter flow cytometry (FCM), quantitative PCR, and detection of donor-recipient chimerism status. For cases without specific fusion gene markers, WT1 gene was sequentially tested with the positive threshold set at 0.6% (1.5% for children). In patients with specific fusion genes such as RUNX1/RUNX1T1 and CBFβ-MYH11, corresponding fusion genes will be monitored. A change from negative to positive or a continuous increase in copy number of the fusion gene is considered indicative of a high risk of relapse. 3-log MRD CBF fusion (RUNX1/RUNX1T1 and CBFβ-MYH11) transcripts reduction can be used to discriminate high-risk from low-risk patients. For patients with detectable MRD, a retest is recommended within two weeks. Bone marrow blasts before transplantation is used to describe disease stage. The group of bone marrow blasts before transplant less than 5% contains AML with complete remission status and MDS patients with blasts less than 5%. Others will be divided into the group with blasts over 5%. The observed indicators included neutrophil and platelet engraftment, acute and chronic graft-versus-host disease (GVHD), grade II-IV acute GVHD (aGVHD), cytomegalovirus (CMV) or Epstein-Barr virus (EBV) reactivation, overall survival (OS), disease-free survival (DFS), relapse rate (RR), and GVHD-free relapse-free survival (GRFS). For neutrophil engraftment, we defined it as a neutrophil count exceeding 0.5*10^9/L for three consecutive days. Similarly, platelet engraftment was defined as a platelet count greater than 20*10^9/L for seven consecutive days without requiring platelet transfusion. The diagnosis and grading of acute and chronic GVHD were based on established recommendations and guidelines (14, 16). Acute GVHD was defined and graded according to Glucksberg-Seattle criteria (GSC) involving three organs (skin, liver and gastrointestinal tract) on four scales (17). Chronic GVHD was evaluated referring to criteria of National Institutes of Health (NIH) (18). In this study, we defined relapse as the occurrence of bone marrow blasts exceeding 5% or the presence of extramedullary lesions after achieving remission. Non-relapse mortality (NRM) was characterized as death during the remission period without experiencing relapse. Overall survival (OS) was determined as the time from transplantation until death or missing data, while disease-free survival (DFS) was calculated from the time of transplant until disease relapse or death. GRFS was defined as survival without relapse and grade III–IV acute GVHD (aGVHD) and chronic GVHD (cGVHD) needing immunosuppressive treatment (11).

### 2.5 Statistical analysis

Data analysis was performed using SPSS version 27 and STATA version 17. Categorical variables were analyzed using the χ2 statistic, while continuous variables were analyzed using the Mann-Whitney test and analysis of variance (ANOVA). For binary variable comparison, both univariate and multivariate Logistic Regression were employed. To compare the cumulative incidence of GVHD, relapse, and NRM, competing risk regression models were utilized. Competing events for GVHD included death or relapse, for NRM it was relapse, and for relapse it was NRM. The Kaplan-Meier method was used to plot OS and DFS curves, and intergroup survival comparison was evaluated using the log-rank test. Cox regression analysis was applied to adjust for any potential biases in survival analysis. Statistical significance was defined as a p-value less than 0.05.

## 3 Results

### 3.1 Baseline characteristics

AML/MDS patients who underwent allo-HSCT at our center from January 2010 to January 2023 were screened for the trial. Data of 251 patients among them were collected and analyzed, and we found there was an imbalance of age (P<0.001), measurable residual disease (MRD) (P=0.045), and CD34+ cell counts (P=0.025) between the two groups (**Table 1**). To overcome the significant differences and make the baseline characteristics of the groups more comparable, we introduced PSM for selection and matched patients at a ratio of 1:1 from aspects of gender, age, diagnosis, donor type, conditioning regimen, and bone marrow blast before transplantation with 0.1 as a caliper width. After the matching course at a ratio of 1:1, 100 pairs (200 patients) were involved in the final trial with no significant difference in the baseline characteristics from aspects of age, gender, diagnosis, HCT-CI, months before transplantation, bone marrow blasts percentage before transplantation, MRD, donor type, graft source, mononuclear, and CD34+ cell counts, ABO compatibility between donor and recipient, conditioning regimen and donor/recipient gender (**Table 1**).

**Table 1:** Baseline characteristics of patients of DAC and non-DAC groups.

	Before PSM			After PSM		
	DAC (n=125)	Non-DAC (n=126)	P value	DAC (n=100)	Non-DAC (n=100)	P value
Age (years)	44(14-65)	36(2-62)	<0.001	41.5(14-65)	38(8-62)	0.102
Gender, n Male Female	68 57	71 55	0.756	59 41	56 44	0.668
WHO classification, n MDS t-AML AML	24 28 73	36 19 71	0.126	20 19 61	19 15 66	0.707
HCT-CI, n 0-2 ≥3	64 61	69 57	0.672	53 47	55 45	0.777
Risk stratification, n			0.585			0.066
Low Relative-intermediate	34 41	22 34		33 29	15 28	
Relative-high	50	46		38	41	
Months before transplant, n			0.525			0.071
<12 months ≥12 months	105 20	102 24		93 7	85 15	
Bone marrow blasts before transplantation, n			0.736			0.861
≤5%	96	99		79	80	
>5%	29	27		21	20	
MRD, n Positive Negative	85 40	99 26	0.045	73 24	78 21	0.557
Type of donor, n MSD-HSCT Haplo-HSCT MUD-HSCT	21 92 12	21 92 13	0.982	15 73 12	16 73 11	0.963
Graft source, n BM+PB PB/BM	103 22	93 33	0.100	82 18	76 24	0.298
MNC (10^8^/kg)	9.8(2-41)	10.4(2-27)	0.059	9.88(2-41)	10.25(2-27)	0.431
CD34+ (10^6^/kg)	5.2(2-20)	4.4(0-17)	0.025	4.83(2-14)	4.75(1-17)	0.504
ABO compatibility, n Matched Minor mismatched Major mismatched	64 24 36	79 19 26	0.152	51 22 26	57 18 24	0.670
Conditioning regimen Bu/Flu Bu/Cy	102 23	99 27	0.325	78 22	81 19	0.599
Donor/recipient gender			0.563			0.837
Female to Female	18	23		14(14%)	17(17.3%)	
Female to Male	28	22		24(24%)	19(19.4%)	
Male to Male	41	44		36(36%)	36(36.7%)	
Male to Female	38	31		26(26%)	26(26.5%)	

DAC, decitabine intensified conditioning regimen; non-DAC, conditioning regimen without decitabine; PSM, propensity score matching; MDS, myeloid dysplastic syndrome; t-AML, transformed acute myeloid leukemia; AML, acute myeloid leukemia; HCT-CI, hematopoietic cell transplantation-specific comorbidity index; MRD, measurable residual disease; HSCT, hematopoietic stem cell transplantation; MSD, matched sibling donor; Haplo, haploidentical donor; MUD, matched unrelated donor; BM, bone marrow; PB, peripheral blood; MNC, mononuclear cell; Bu, busulfan; Flu, fludarabine; Cy, cyclophosphamide.

### 3.2 Engraftment and engraftment time

The neutrophil engraftment rate was 98% in the DAC group and 100% in the non-DAC group. Similarly, 93 patients in the DAC group and 99 patients in the non-DAC group reached platelet engraftment. The comparison of neutrophil and platelet engraftment rates between the two groups showed no statistical difference (P=0.497 and 0.071, respectively). The median time for neutrophil and platelet engraftment was 12 (1-25) and 17 (1-180) days, respectively, in the DAC group, while in the non-DAC group, it was 12 (9-29) and 15 (6-270) days, respectively. The hematopoietic engraftment period was found to be comparable between the two groups (P=0.893 and 0.178 for neutrophil and platelet engraftment, respectively). Donor chimerism was tested by day 30 to determine the successful transplant. Donor chimerism over 95% was defined as full donor chimerism. In this cohort, 98 patients in non-DAC group got full donor chimerism by day 30, and donor chimerism of the other two patients was 94% and 92% respectively. For DAC group, 97 patients got full donor chimerism, and the other three patients died within one month after HSCT without chimerism detection. We compared the rate of full donor chimerism by day 30 of the patients, and there was no significant difference between the two groups(P=0.498). **Table 2** presents these results.

**Table 2:** Engraftment data of DAC and non-DAC groups.

	DAC (n=100)	Non-DAC (n=100)	P value
NE engraftment	98	100	0.497
PLT engraftment	93	99	0.071
Median engraftment time of NE	12 (1-25)	12 (9-29)	0.893
Median engraftment time of PLT	17 (1-180)	15 (6-270)	0.178
Full donor chimerism by day 30	97	98	0.498

DAC, decitabine intensified conditioning regimen; non-DAC, conditioning regimen without decitabine; NE, neutrophil; PLT, platelet.

### 3.3 Complications after HSCT

**Figure 1:**
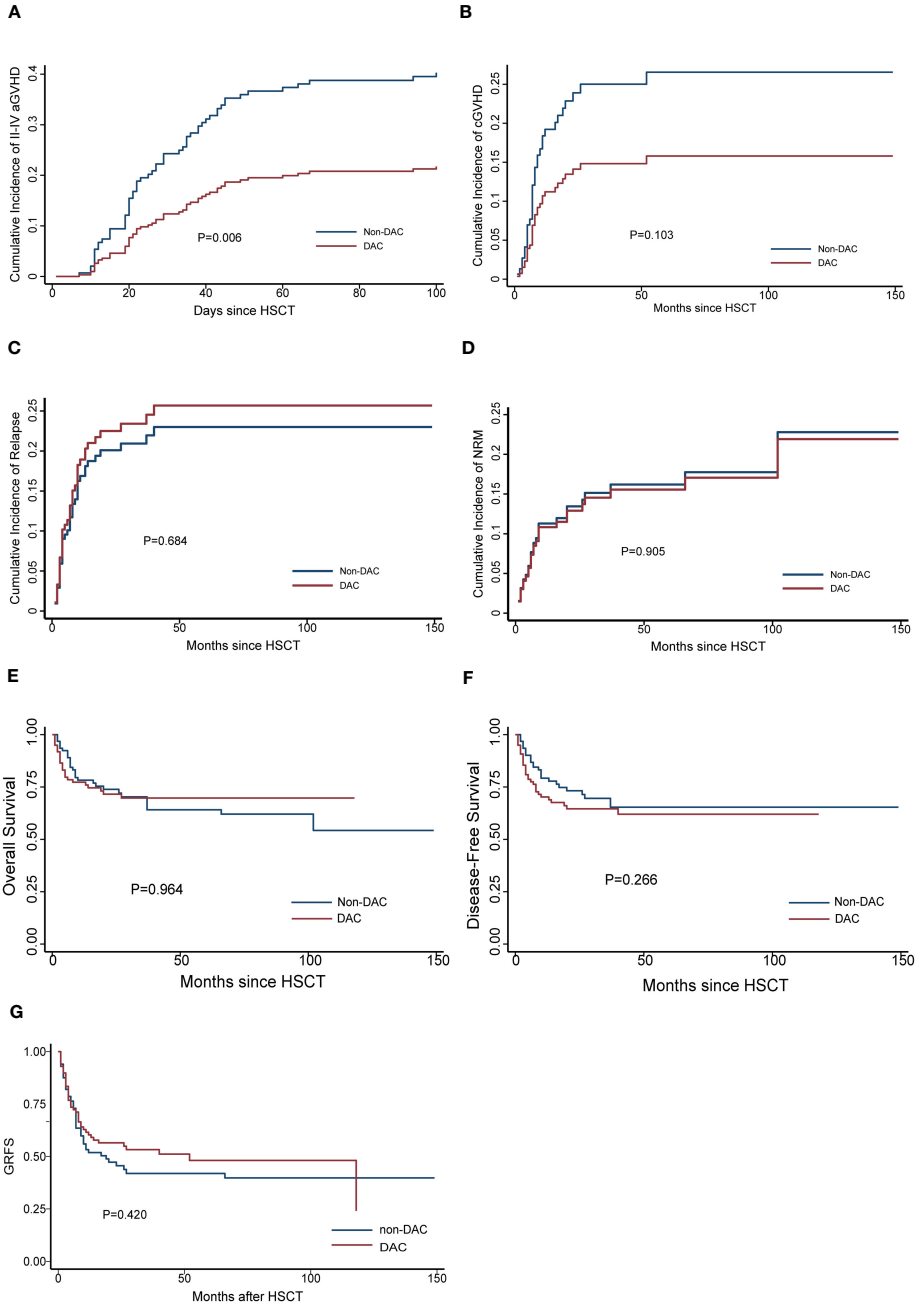
Cumulative GVHD (**A** for grade II-IV aGVHD, **B** for cGVHD), **(C)** relapse, **(D)** NRM, **(E)** OS, **(F)** DFS and **(G)** GRFS for the entire population after PSM. DAC, decitabine intensified conditioning regimen; non-DAC, conditioning regimen without decitabine; aGVHD, acute graft versus host disease; cGVHD, chronic graft versus host disease; NRM, non-relapse mortality; HSCT, hematopoietic stem cell transplantation; GRFS: GVHD free, relapse free survival.

We conducted a comparison of HSCT-related complications between the DAC and non-DAC groups. The overall incidence of acute GVHD was 38% in the DAC group and 47% in the non-DAC group, showing no significant difference (P=0.198). However, our further analysis revealed that the incidence of grade II-IV acute GVHD (aGVHD) in the DAC group was significantly lower at 21%, compared to 38% in the non-DAC group (P=0.013) (**Table 3**). While there was a tendency that patients in DAC group experienced lower incidence of grade III-IV aGVHD (P=0.085). We conducted univariate analysis involving age, gender, diagnosis, bone marrow blasts, measurable residual disease, conditioning regimen, donor type, graft type, mononuclear cell counts, and ABO compatibility. Univariate logistic analyses indicated that the administration of decitabine was a protective factor against grade II-IV aGVHD (P=0.017, OR=0.473, 95% CI 0.231-0.813). Multivariate logistic analysis indicated that decitabine remained a protective factor against grade II-IV aGVHD (P=0.018, OR=0.460, 95% CI 0.242-0.875), and haplo-identical donor was a risk factor for grade II-IV aGVHD in comparison to matched sibling donor (P=0.027, OR=4.124, 95% CI 1.175-14.473). The incidence of chronic GVHD (cGVHD) tended to be lower in the DAC group, but the difference did not reach statistical significance (P=0.054). For severe cGVHD, the between-group difference was not significant (P=0.212). We further analyzed the moderate to severe cGVHD and found that moderate to severe cGVHD tended to decrease in DAC group (**Table 3**). We further conducted competing risk regression analysis (**Tables 4**, **5**). As **Figure 1** showed, the incidence of grade II-IV aGVHD was significantly lower in the DAC group (P=0.006) (**Figure 1**). Univariate competing analysis showed DAC was a protective factor against grade II-IV GVHD (P=0.006, SHR=0.5, 95% CI 0.3-0.84). Female and conditioning regimen with Bu/Flu were protective factors against cGVHD (P=0.01, SHR=2.93, 95% CI 1.29-6.71; P=0.01, SHR=2.57, 95% CI 1.28-5.14, respectively) (**Table 4**). It was noted that in univariate analysis for grade II-IV aGVHD none of the factors had a P-value below 0.1, except for the administration of DAC. This observation held true in both the univariate logistic regression and the univariate competing risk regression. As a result, we opted to incorporate all potential influencing factors, including age, gender, diagnosis, risk stratification, time elapsed before transplantation, bone marrow blast count before HSCT, MRD status, donor type, graft source, CD34+ cell count, ABO compatibility, conditioning regimen, and the administration of DAC, into the subsequent multivariate analysis. As multivariate competing regression analysis showed in **Table 5**, administration of decitabine was again confirmed as a protective factor against grade II-IV aGVHD (P=0.038, SHR=0.53, 95% CI 0.29-0.97). Male patients (P=0.03, SHR=2.78, 95% CI 1.11-6.97) and those receiving a Bu/Cy-based conditioning regimen (P=0.01, SHR=2.90, 95% CI 1.28-6.61) showed an increased likelihood of cGVHD occurrence. However, the decitabine-containing regimen did not influence other complications, including CMV and EBV reactivation, pneumonia, and hemorrhagic cystitis (P>0.05) (**Table 3**).

**Table 3:** Complications of DAC and non-DAC group.

	DAC (n=100)	Non-DAC (n=100)	P value
Acute GVHD	38	47	0.198
Grade II-IV° acute GVHD	21	38	0.013
Grade III-IV° acute GVHD	3	10	0.085
Chronic GVHD	11	21	0.054
Severe chronic GVHD	1	5	0.212
Moderate to severe cGVHD	2	9	0.063
CMV	65	65	1.000
EBV	42	38	0.564
Pneumonia	47	52	0.479
Hemorrhagic cystitis	28	32	0.537

DAC, decitabine intensified conditioning regimen; non-DAC, conditioning regimen without decitabine; GVHD, graft versus host disease; CMV, cytomegalovirus; Epstein-Barr virus.

**Table 4:** Univariate competing risk regression for cumulative incidence of GVHD, CIR, NRM.

Variable	Grade II-IV aGVHD	cGVHD	Relapse	NRM
	SHR (95% CI) P	SHR (95% CI) P	SHR (95% CI) P	SHR (95% CI) P
Age	0.97(0.96-1.01) 0.18	0.97(0.95-1.00) 0.07	1.00(0.98-1.02) 0.68	1.02(0.98-1.06) 0.19
Gender	1.25(0.74-2.12) 0.40	2.93(1.29-6.71) 0.01	2.01(1.01-4.01) 0.05	1.27(0.58-2.76) 0.55
WHO classification	1.15(0.81-1.62) 0.45	1.09(0.67-1.79) 0.71	0.97(0.67-1.40) 0.88	0.92(0.58-1.47) 0.73
Risk stratification	1.14(0.82-1.58) 0.41	0.81(0.53-1.24) 0.33	1.02(0.69-1.53) 0.60	1.44(0.85-2.45) 0.18
Months before transplantation	1.49(0.71-3.13) 0.29	0.53(0.12-2.28) 0.40	1.33(0.51-3.44) 0.56	1.50(0.52-4.32) 0.45
Bone marrow blasts before transplantation	0.79(0.45-1.39) 0.38	1.34(0.58-3.11) 0.50	0.51(0.27-0.99) 0.05	0.35(0.17-0.72) 0.004
MRD	1.59(0.69-3.67) 0.42	0.83(0.37-1.83) 0.64	2.03(0.79-5.21) 0.14	1.29(0.50-3.34) 0.59
Type of donor	0.97(0.67-1.41) 0.88	0.98(0.49-1.99) 0.97	0.71(0.37-1.32) 0.28	1.33(0.77-2.31) 0.30
Graft source	0.99(0.53-1.84) 0.97	1.58(0.55-4.58) 0.40	0.94(0.45-1.94) 0.86	2.23(0.76-6.53) 0.14
CD34+ cells	1.06(0.99-1.14) 0.12	0.98(0.85-1.12) 0.76	0.99(0.88-1.12) 0.86	1.03(0.90-1.18) 0.63
ABO compatibility	1.07(0.81-1.43) 0.62	0.99(0.67-1.48) 0.98	0.96(0.67-1.39) 0.84	0.91(0.60-1.38) 0.67
Conditioning regimen	1.10(0.60-2.01) 0.60	2.57(1.28-5.14) 0.01	0.85(0.38-1.91) 0.71	1.40(0.60-3.27) 0.43
DAC	0.50(0.30-0.84) 0.006	0.56(0.27-1.13) 0.103	1.14(0.62-2.10) 0.68	0.96(0.46-1.98) 0.91

DAC, decitabine intensified conditioning regimen; aGVHD, acute graft versus host disease; cGVHD, chronic graft versus host disease; NRM, non-relapse mortality; CIR, cumulative incidence of relapse; MRD, measurable residual disease.

**Table 5:** Multivariate competing risk regression for cumulative incidence of GVHD, CIR, NRM.

Variable	Grade II-IV aGVHD	cGVHD	Relapse	NRM
	SHR (95% CI) P	SHR (95% CI) P	SHR (95% CI) P	SHR (95% CI) P
Age	0.98(0.96-1.00) 0.11	0.97(0.96-1.00) 0.09	1.01(0.98-1.03) 0.59	1.02(0.98-1.06) 0.30
Gender	1.15(0.63-2.11) 0.76	2.78(1.11-6.97) 0.03	2.45(0.97-6.21) 0.06	0.98(0.35-2.71) 0.97
WHO classification	1.04(0.67-1.62) 0.85	0.78(0.43-1.40) 0.40	1.36(0.87-2.11) 0.12	1.18(0.61-2.31) 0.62
Risk stratification	1.09(0.76-1.59) 0.63	0.86(0.51-1.46) 0.57	0.89(0.58-1.36) 0.60	1.33(0.75-2.39) 0.32
Months before transplantation	1.34(0.54-3.31) 0.53	0.66(0.09-4.86) 0.69	1.41(0.33-6.09) 0.65	1.31(0.42-4.12) 0.64
Bone marrow blasts before transplantation	0.74(0.39-1.43) 0.38	1.24(0.46-3.36) 0.68	0.54(0.24-1.23) 0.14	0.32(0.11-0.88) 0.028
MRD	1.59(0.69-3.67) 0.28	0.52(0.21-1.29) 0.16	1.56(0.59-4.10) 0.37	0.55(0.29-2.95) 0.90
Type of donor	1.45(0.69-3.07) 0.33	1.26(0.44-3.68) 0.66	0.57(0.27-1.18) 0.13	2.90(0.63-13.24) 0.17
Graft source	2.13(0.66-6.87) 0.21	1.58(0.55-4.58) 0.40	0.55(0.19-1.57) 0.26	3.94(0.73-21.35) 0.11
CD34+ cells	1.04(0.96-1.13) 0.32	0.92(0.76-1.11) 0.39	1.01(0.90-1.14) 0.84	1.03(0.89-1.19) 0.71
ABO compatibility	0.92(0.65-1.30) 0.64	0.96(0.64-1.45) 0.85	1.03(0.70-1.54) 0.87	0.92(0.60-1.42) 0.72
Conditioning regimen	0.82(0.37-1.78) 0.61	2.90(1.28-6.61) 0.01	0.68(0.26-1.75) 0.42	0.68(0.24-1.93) 0.47
DAC	0.53(0.29-0.97) 0.038	0.54(0.22-1.33) 0.183	0.99(0.49-2.01) 0.98	1.05(0.47-2.35) 0.90

DAC, decitabine intensified conditioning regimen; aGVHD, acute graft versus host disease; cGVHD, chronic graft versus host disease; NRM, non-relapse mortality; CIR, cumulative incidence of relapse; MRD, measurable residual disease.

### 3.4 RR, NRM, OS and DFS

The median follow-up time for the entire population, DAC group, and non-DAC group were 18.5 months (ranging from 0 to 149 months), 18 months (ranging from 0 to 118 months), and 19.5 months (ranging from 1 to 149 months), respectively. The cumulative incidence of relapse and non-relapse mortality (NRM) showed no significant difference between the two groups (**Figure 1**, P=0.684 and 0.905, respectively). In our univariate and multivariate analysis (**Tables 4**, **5**), we identified bone marrow blasts >5% before transplantation as an independent risk factor for NRM (P=0.004, SHR=0.35, 95% CI 0.17-0.72; P=0.028, SHR=0.32, 95%CI 0.11-0.88). The 2-year overall survival (OS) rate for the DAC and non-DAC groups was 71.6% and 73.9%, respectively. The 2-year disease-free survival (DFS) rate for the DAC group and non-DAC group was 64.6% and 73.2%, respectively. The 2-year GVHD free, relapse free survival (GRFS) rate for the DAC group and non-DAC group was 56.5% and 45.6%, respectively. However, the Kaplan-Meier curves showed no significant difference in OS and DFS between the two groups (P= 0.964, 0.266 and 0,420 for OS, DFS, and GRFS respectively). Furthermore, we conducted univariate cox regression and found male and blast before HSCT less than 5% tended to survive longer (P=0.04, SHR=1.84, 95% CI 1.03-3.3; P<0.001, SHR= 0.29, 95% CI 0.17-0.50) (**Table 6**). Lower blasts before HSCT tended to extend DFS and GRFS as well (P=0.001, SHR=0.4, 95% CI 0.23-0.68; P=0.02, SHR 0.58, 95%CI 0.36-0.9). Male patients tended to have longer GRFS (P=0.001, SHR=2.1, 95%CI 1.33-3.39), which was confirmed in multivariate analysis (P=0.002, SHR=2.21, 95%CI 1.32-3.71). In multivariate analysis (**Table 7**), we found that bone marrow blasts over 5% before transplant remained an independent risk factor for poor overall survival and disease-free survival (P<0.001, SHR=0.25, 95%CI 0.12-0.52 and P=0.004, SHR=0.36, 95%CI 0.18-0.72, respectively). Additionally, compared with MDS and transformed AML, a diagnosis of AML was a risk factor for poor disease-free survival (P=0.04, SHR=1.58, 95%CI 1.03-2.43).

**Table 6:** Univariate cox regression for OS and DFS.

Variable	OS	DFS	GRFS
	SHR (95% CI) P	SHR (95% CI) P	SHR (95% CI) P
Age	1.02(0.99-1.04) 0.15	1.01(0.99-1.03) 0.26	1.00(0.99-1.02) 0.01
Gender	1.84(1.03-3.30) 0.04	1.52(0.88-2.65) 0.13	2.10(1.33-3.29) 0.001
WHO classification	0.90(0.65-1.25) 0.53	1.04(0.75-1.45) 0.80	1.03(0.79-1.33) 0.85
Risk stratification	1.54(1.05-2.26) 0.03	1.10(0.78-1.54) 0.59	1.17(0.89-1.53) 0.26
Months before transplantation	1.55(0.73-3.30) 0.25	1.55(0.74-3.27) 0.25	1.28(0.68-2.41) 0.44
Bone marrow blasts before transplantation	0.29(0.17-0.50) <0.001	0.40(0.23-0.68) 0.001	0.58(0.36-0.90) 0.02
MRD	1.68(0.79-3.57) 0.18	1.65(0.81-3.36) 0.17	1.63(0.94-2.86) 0.08
Type of donor	1.06(0.64-1.74) 0.83	0.84(0.51-1.37) 0.48	0.86(0.58-1.28) 0.47
Graft source	1.44(0.73-2.84) 0.30	1.33(0.70-2.53) 0.39	1.31(0.79-2.15) 0.28
CD34+ cells	1.02(0.93-1.12) 0.50	1.02(0.93-1.11) 0.73	1.03(0.96-1.10) 0.46
ABO compatibility	0.90(0.66-1.23) 0.51	0.98(0.72-1.31) 0.87	1.02(0.80-1.30) 0.86
Conditioning regimen	1.06(0.55-2.05) 0.87	1.07(0.57-2.04) 0.82	0.15(0.91-2.35) 0.11
DAC	1.01(0.59-1.72) 0.97	1.34(0.80-2.24) 0.27	0.84(0.56-1.27) 0.42

**Table 7:** Multivariate cox regression for OS and DFS.

Variable	OS	DFS	GRFS
	SHR (95% CI) P	SHR (95% CI) P	SHR (95% CI) P
Age	1.01(0.99-1.04) 0.32	1.01(0.99-1.04) 0.22	0.99(0.98-1.01) 0.87
Gender	1.73(0.89-3.37) 0.11	1.78(0.94-3.78) 0.08	2.21(1.32-3.71) 0.002
WHO classification	1.25(0.81-1.93) 0.30	1.58(1.03-2.43) 0.04	1.15(0.83-1.60) 0.39
Risk stratification	1.44(0.96-2.15) 0.08	1.01(0.70-1.44) 0.98	1.12(0.84-1.50) 0.41
Months before transplantation	1.59(0.62-4.09) 0.33	1.98(0.79-4.94) 0.14	1,27(0.57-2.86) 0.56
Bone marrow blasts before transplantation	0.25(0.12-0.52) <0.001	0.36(0.18-0.72) 0.004	0.59(0.34-1.04) 0.07
MRD	0.95(0.40-2.27) 0.91	1.07(0.49-2.41) 0.86	1.09(0.59-2.01) 0.78
Type of donor	1.33(0.60-2.94) 0.48	0.87(0.44-1.73) 0.70	0.99(0.57-1.74) 0.98
Graft source	2.01(0.67-6.07) 0.21	1.14(0.44-2.94) 0.79	1.23(0.59-2.56) 0.56
CD34+ cells	1.05(0.95-1.16) 0.32	1.05(0.96-1.16) 0.30	1.03(0.96-1.12) 0.35
ABO compatibility	0.91(0.65-1.27) 0.57	1.03(0.74-1.44) 0.84	1.02(0.78-1.32) 0.86
Conditioning regimen	0.59(0.25-1.39) 0.23	0.67(0.31-1.47) 0.32	0.99(0.55-1.76) 0.96
DAC	1.08(0.60-1.96) 0.80	1.34(0.73-2.45) 0.34	0.89(0.56-1.41) 0.62

OS, overall survival; DFS, disease-free survival; DAC, decitabine intensified conditioning regimen; MRD, measurable residual disease.

### 3.5 Subgroup analysis

**Figure 2:**
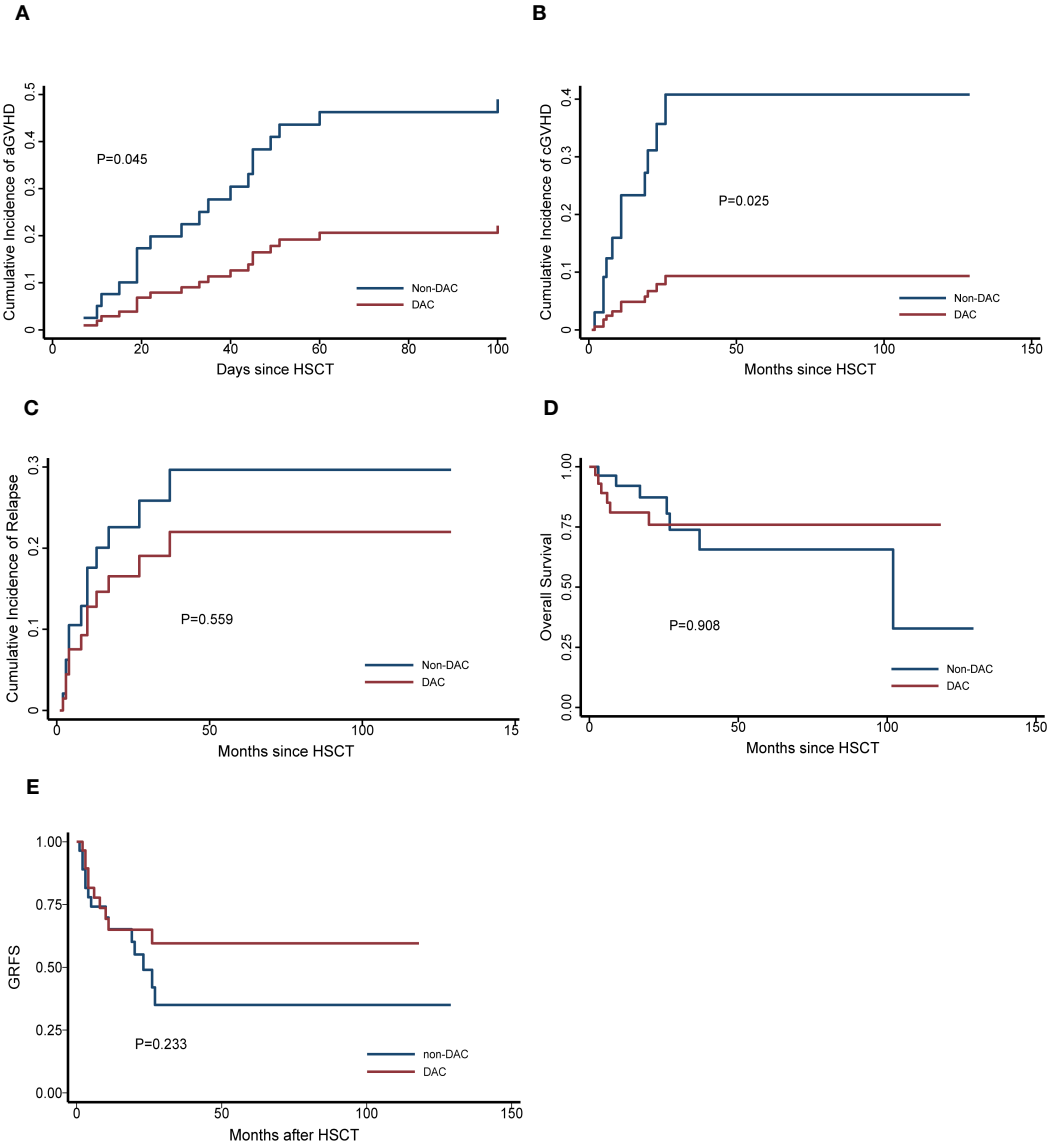
Outcomes in patients divided into relatively intermediate risk subgroup. **(A)** Cumulative incidence of grade II-IV aGVHD, **(B)** Cumulative incidence of cGVHD, **(C)** Cumulative incidence of relapse, **(D)** Overall survival, **(E)** GRFS. DAC, decitabine intensified conditioning regimen; non-DAC, conditioning regimen without decitabine; HSCT hematopoietic stem cell transplantation; aGVHD, acute graft versus host disease; cGVHD, chronic graft versus host disease; GRFS: GVHD free, relapse free survival.

**Figure 3:**
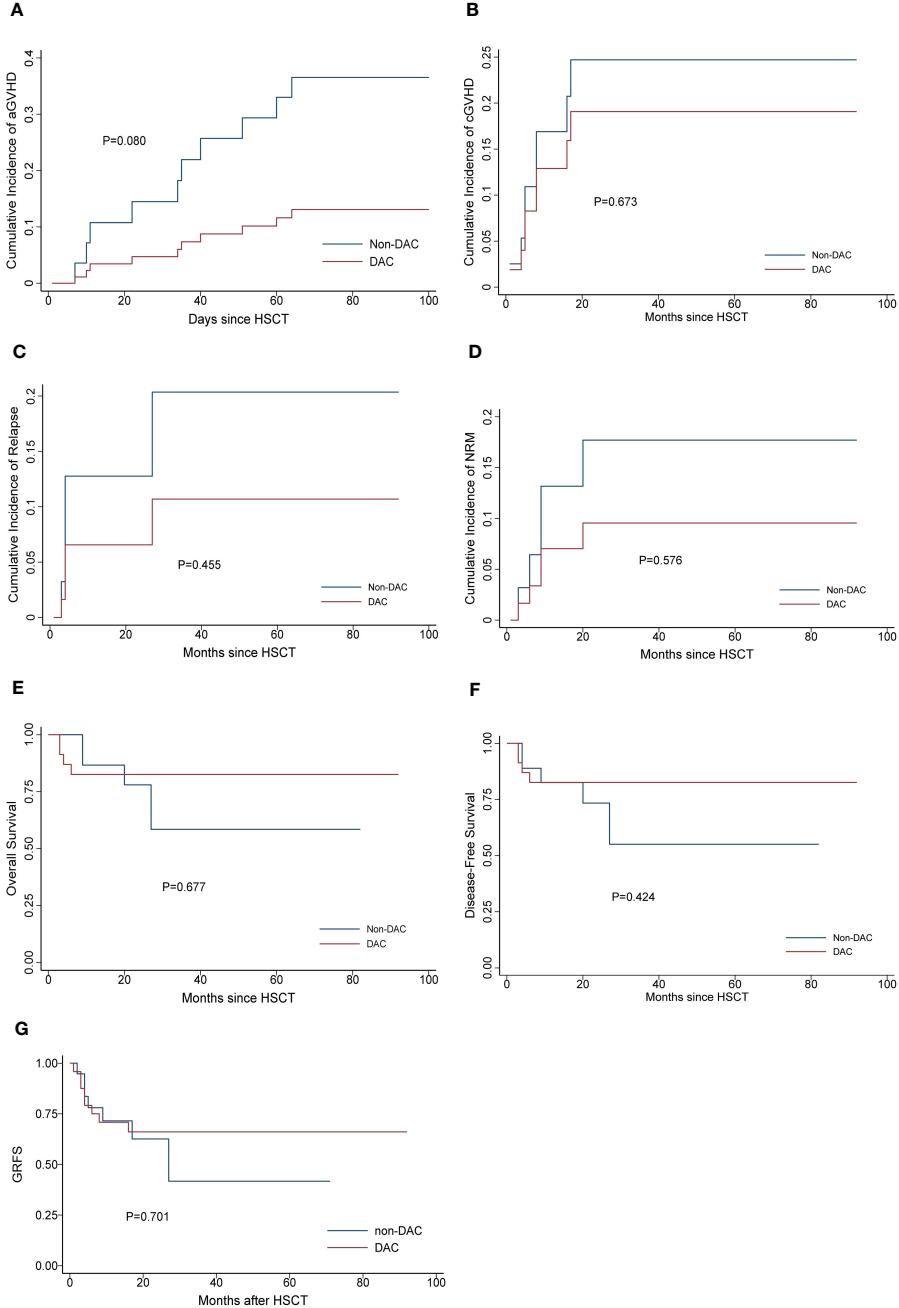
Outcomes in patients divided into MRD negative subgroup. **(A)** Cumulative incidence of grade II-IV aGVHD, **(B)** Cumulative incidence of cGVHD, **(C)** Cumulative incidence of relapse. **(D)** Cumulative incidence of NRM, **(E)** Overall survival, **(F)** Disease-free survival, **(G)** GRFS. DAC, decitabine intensified conditioning regimen; non-DAC, conditioning regimen without decitabine; HSCT hematopoietic stem cell transplantation; aGVHD, acute graft versus host disease; cGVHD, chronic graft versus host disease; NRM, non-relapse mortality; GRFS: GVHD free, relapse free survival.

To further investigate the effect of DAC-containing regimens on different subgroups, we stratified patients based on MRD status and risk stratification. In the relatively intermediate risk subgroups, which included MDS patients with IPSS-R scores ranging from 3.5 to 6 and AML patients considered to have intermediate cytogenetic risk, we observed a lower cumulative incidence of grade II-IV acute GVHD (aGVHD) and chronic GVHD (cGVHD) in patients receiving DAC-containing conditioning regimens (P=0.045 and 0.025, respectively). Additionally, the DAC subgroup tended to have a lower cumulative incidence of relapse, longer overall survival and GRFS, although these differences were not statistically significant (P=0.559, 0.908 and 0.233, respectively) (**Figure 2**). In the MRD negative subgroup, patients receiving DAC-containing regimens showed a tendency towards lower incidences of grade II-IV aGVHD, cGVHD, relapse, and non-relapse mortality (NRM), as well as longer survival and GRFS (**Figure 3**). However, the differences were not statistically significant in this subgroup.

## 4 Discussion

Decitabine, a DNA methyltransferase suppressor, has emerged as a promising anti-tumor drug due to its ability to reactivate silenced tumor suppressor genes and act as a cytotoxic agent in synergy with busulfan. Nowadays, DAC has been widely utilized in the therapy of hematopoietic malignancies. First, DAC is particularly beneficial for patients who cannot tolerate standard-dose chemotherapy but still aim to achieve remission, owing to ideal antitumor effect and relatively low toxicity. Second, incorporating DAC into consolidation therapy, before-HSCT bridging therapy, and maintenance therapy after HSCT has shown improvements in long-term outcomes.

Remarkably, beyond its antitumor effect, the potential immune-regulatory properties of decitabine have garnered even more interest. Low doses of decitabine have been found to promote the polarization of T cells from Th1 type to regulatory T cells (Tregs), regulate T cell proliferation via the TET2 pathway, and suppress the development of proinflammatory cytokines. These immune-modulatory effects could potentially alleviate GVHD by modulating immune tolerance. Moreover, studies in animal models have shown that hypomethylating agents, including decitabine, can alleviate chronic GVHD (19). In our study, patients who received a decitabine-based conditioning regimen experienced a lower incidence of grade II-IV aGVHD and exhibited milder symptoms, leading to a reduction in the cumulative incidence of grade II-IV aGVHD from 38% to 21%. These results align with findings reported by Li and colleagues (11). Moreover, our study population receiving a decitabine-based regimen was larger than that in any other previous studies investigating the impact of such intensified conditioning regimens on post-HSCT prognosis. Furthermore, both multivariate logistic regression and competing risk regression analyses indicated that the administration of decitabine was associated with a decreased risk of grade II-IV aGVHD. These robust findings underscore the reliability of decitabine-containing conditioning regimens in preventing acute GVHD. In summary, our study provides further evidence supporting the potential of decitabine as an effective agent in preventing acute GVHD, in addition to its known antitumor properties. The immune-regulatory effects of decitabine make it a promising candidate for enhancing the outcomes of allogeneic hematopoietic stem cell transplantation.

As mentioned above, the function of DAC varies with the dose used. High-dose DAC acts as an antitumor agent, while low-dose DAC tends to regulate immune responses. To reduce relapse and extend DFS, a relatively higher dose is recommended. However, to also consider the immune regulatory function, daily dose at about 20mg/m^2^ for 5 days is commonly selected, though there is no consensus on the exact dose. For instance, a study by Cao and colleagues demonstrated that MDS/MPN patients receiving 5-day DAC at a daily dose of 20mg/m^2^ had an OS of 86% and a relapse rate of 12% at a median follow-up time of 522 days (20). The beneficial effects were more pronounced in the high-risk subgroup with active disease, indicating that DAC administration might enhance survival and delay relapse (21). A study showed that compared with a total dose of 75mg/m^2^, DAC at a dose of 125mg/m^2^ tended to prolong the DFS (22). In our previous study, we mechanistically demonstrated that low-dose decitabine, when combined with the histone H3K27 methyltransferase inhibitor 3-deazaneplanocin (DZNep), could alleviate aGVHD by promoting T cell differentiation into regulatory T cells in animal model (23). Moreover, we found that compared to 2mg/kg, DAC at a lower dose of 1mg/kg exhibited a reduced risk of cytopenia, further highlighting the safety and immune-regulation effects of low-dose DAC. In this study, we selected a dose of 15mg/m^2^, and significantly, the incidence of grade II-IV acute GVHD (aGVHD) decreased by almost half, highlighting the immune regulatory function of DAC at this lower dose. However, in the MRD positive or high-risk subgroup, the impact of DAC intervention was not as evident, possibly due to the weakened antitumor effect at a lower DAC dose. The precise titration of DAC dose warrants further investigation to strike a balance between its antitumor and immune regulatory effects.

Indeed, Li and colleagues’ study also indicated that a decitabine-containing regimen was associated with reduced cGVHD. In our study, we compared the occurrence of cGVHD between the two groups and observed a tendency towards a lower incidence of cGVHD in the DAC group. Notably, in the intermediate-risk subgroup, patients receiving DAC exhibited a significantly lower incidence of cGVHD These findings suggest that decitabine may have a potential role in alleviating cGVHD, particularly in intermediate-risk patients. However, to draw an exact conclusion, further investigation and validation are necessary.

During the follow-up period, we carefully assessed the influence of DAC on overall survival. In the analysis of the entire study population, the differences between the groups were not prominently apparent. However, when we conducted a more detailed investigation by refining the subgroups, certain trends became evident. Specifically, in the MRD negative subgroup and intermediate risk subgroup, patients who received DAC-containing regimens showed a tendency towards improved relapse rates and overall survival. Moreover, in the MRD negative patients, DAC appeared to positively impact NRM and DFS as well. These findings underscore the potential benefits of incorporating DAC into the treatment approach for MRD negative or intermediate risk patients. By reducing the risk of relapse and enhancing overall survival, DAC-containing regimens hold promise as a valuable therapeutic option for these specific patient groups.

Our study revealed that having bone marrow blasts less than 5% before transplant was a protective factor associated with lower NRM and improved OS and DFS. This finding aligns with existing literature, emphasizing the significance of achieving complete remission status in determining post-transplant prognosis. Recently, MRD has garnered significant attention, as studies have shown it to be a risk factor for relapse, and eliminating MRD has been associated with longer survival (24, 25). Our subgroup analysis demonstrated that in the MRD negative subgroup, the administration of DAC might benefit the patients. However, in the MRD positive subgroup, DAC alone could not fully overcome the adverse effect of MRD. This underscores the critical importance of MRD elimination before transplant, and in such cases, the administration of DAC might have a more significant impact.

In theory, DAC, with its ability to target abnormal methylation processes, holds promise for improving the prognosis of high cytogenetic risk populations with specific mutations, such as TP53. Several studies have observed that DAC can enhance the survival of high-risk patients (20, 26). However, in our study, we found that the beneficial effect of DAC was more pronounced in the intermediate risk subgroup than in the high-risk subgroup. Therefore, it would be meaningful to conduct future research specifically targeting high-risk patients at our center.

Importantly, concerns have been raised by some researchers regarding the potential increase in infection incidence when combining DAC induction with myeloablative regimens (27). However, in our study, the addition of DAC did not result in any additional infectious toxicity or impaired engraftment. This finding highlights the favorable safety profile of the conditioning regimen protocol with additive DAC.

Though the results revealed grade II-IV aGVHD decreased in DAC group, I think it is far from drawing an exact conclusion DAC ameliorate grade II-IV aGVHD. Nonetheless, it is crucial to acknowledge the limitations inherent in our study. The retrospective, single-center nature of our research inherently contributes to a lower level of evidence certainty. We are aware that information bias could have arisen during the data collection process. The extended duration of the study may have resulted in the withdrawal of some patients, introducing attrition bias. Furthermore, the heterogeneity within the study population may have introduced various biases as well. While we recognize that our study falls short of delivering a definitive conclusion, it still holds value as a source of inspiration. To address these limitations and provide a more robust understanding of the topic, we are currently conducting a single-center Randomized Controlled Trial (RCT). We aim to expand the scale of the study, involve multiple centers, and follow a prospective design to rigorously investigate whether DAC can effectively mitigate grade II-IV aGVHD in clinical practice.

## 5 Conclusion

In conclusion, our analysis of AML/MDS patients undergoing allo-HSCT with a low dose DAC-containing conditioning regimen revealed its efficacy in ameliorating grade II-IV acute GVHD. Notably, in MRD negative or intermediate risk patients, this regimen showed promise in preventing relapse and improving survival without added toxicity. However, it is important to acknowledge the limitations of our study, including its retrospective nature and the need for further prospective exploration to determine its potential benefits for high-risk and MRD positive patients.

## Data availability statement

The original contributions presented in the study are included in the article/supplementary material. Further inquiries can be directed to the corresponding author.

## Ethics statement

The studies involving humans were approved by the Review Board of Peking University First Hospital. The studies were conducted in accordance with the local legislation and institutional requirements. Written informed consent for participation was not required from the participants or the participants’ legal guardians/next of kin in accordance with the national legislation and institutional requirements.

## Author contributions

YL: Conceptualization, Funding acquisition, Methodology, Writing – review & editing. JZ: Data curation, Formal Analysis, Investigation, Writing – original draft. QYaW: Data curation, Formal Analysis, Investigation, Writing – original draft. HR: Data curation, Resources, Writing – review & editing. YD: Data curation, Resources, Writing – review & editing. YY: Data curation, Resources, Writing – review & editing. QW: Data curation, Resources, Writing – review & editing. ZL: Data curation, Resources, Writing – review & editing. WL: Data curation, Resources, Writing – review & editing. QYunW: Data curation, Resources, Writing – review & editing. BW: Data curation, Resources, Writing – review & editing.
